# Recurrent right hepatic artery pseudoaneurysm after robotic-assisted cholecystectomy in a patient with Mirizzi syndrome: a case report

**DOI:** 10.1186/s12893-021-01438-2

**Published:** 2022-03-23

**Authors:** Ted Hsiung, Tsung-Shih Lee, Yueh-Lin Lee, Ting-Shuo Huang, Chih-Yuan Wang

**Affiliations:** 1 Department of General Surgery, Keelung Chang Gung Memorial Hospital, Keelung, 204 Taiwan; 2 Division of Hepato-gastroenterology, Keelung Chang Gung Memorial Hospital, Keelung, 204 Taiwan; 3 Department of Radiology, Keelung Chang Gung Memorial Hospital, Keelung, 204 Taiwan

**Keywords:** Iatrogenic hepatic artery pseudoaneurysm, Robotic-assisted surgery, Mirizzi syndrome, Case report

## Abstract

**Background:**

Iatrogenic hepatic artery pseudoaneurysm is a rare complication following laparoscopic cholecystectomy. Trans-arterial embolization (TAE) is an effective way to control bleeding after a ruptured aneurysm. But uncommonly, rebleeding may occur which will require a second embolization or even laparotomy.

**Case presentation:**

We report a case of a 45-year-old woman who underwent robotic-assisted cholecystectomy after the diagnosis of type II Mirizzi syndrome. During the operation, the anterior branch of the right hepatic artery was damaged and Hem-o-lok clips were applied to control the bleeding. The postoperative course was smooth, and the patient was discharged 6 days after the procedure. However, one week after hospital discharge, she presented to the emergency department with right upper abdominal tenderness, melena, and jaundice. After examination, the computed tomography angiography (CTA) revealed a 3 cm pseudoaneurysm at the distal stump of the right hepatic artery anterior branch. TAE with gelfoam material was performed. Three days later, the patient had an acute onset of abdominal pain. A recurrent pseudoaneurysm was found at the same location. She underwent TAE again but this time with a steel coil. No further complication was noted, and she was discharged one week later.

**Conclusions:**

Even with the assistance of modern technologies such as the robotic surgery system, one should still take extra caution while handling the vessels. Also, embolization of the pseudoaneurysm with steel coils may be suitable for preventing recurrence.

## Background

Iatrogenic right hepatic artery pseudoaneurysm is a rare complication after laparoscopic cholecystectomy. The clinical symptoms and signs are mainly attributable to hemobilia and usually happen around 2 weeks after surgery [1]. Early diagnosis with endoscopy and computed tomography angiography following prompt treatment with trans-arterial embolization (TAE) is mandatory. The clinical success rate of TAE is high, although recurrent pseudoaneurysm bleeding after TAE could happen, and repeated TAE or surgical intervention may be needed [2, 3]. The reason for pseudoaneurysm formation may be due to excessive vessel manipulation or thermal injury during the operation, especially in situations of complicated cholecystitis or distorted gall bladder anatomy like Mirizzi syndrome. Robotic-assisted cholecystectomy may facilitate vessel control and prevent inadvertent bile duct injury compared to the traditional laparoscopy approach. However, carefully dealing with the vessel with or without an energy device still needs to be emphasized. Here we present the case of a recurrent iatrogenic right hepatic artery branch pseudoaneurysm after robotic-assisted cholecystectomy following two TAE interventions for the cessation of the bleeding from the pseudoaneurysm.

## Case presentation

**Fig. 1:**
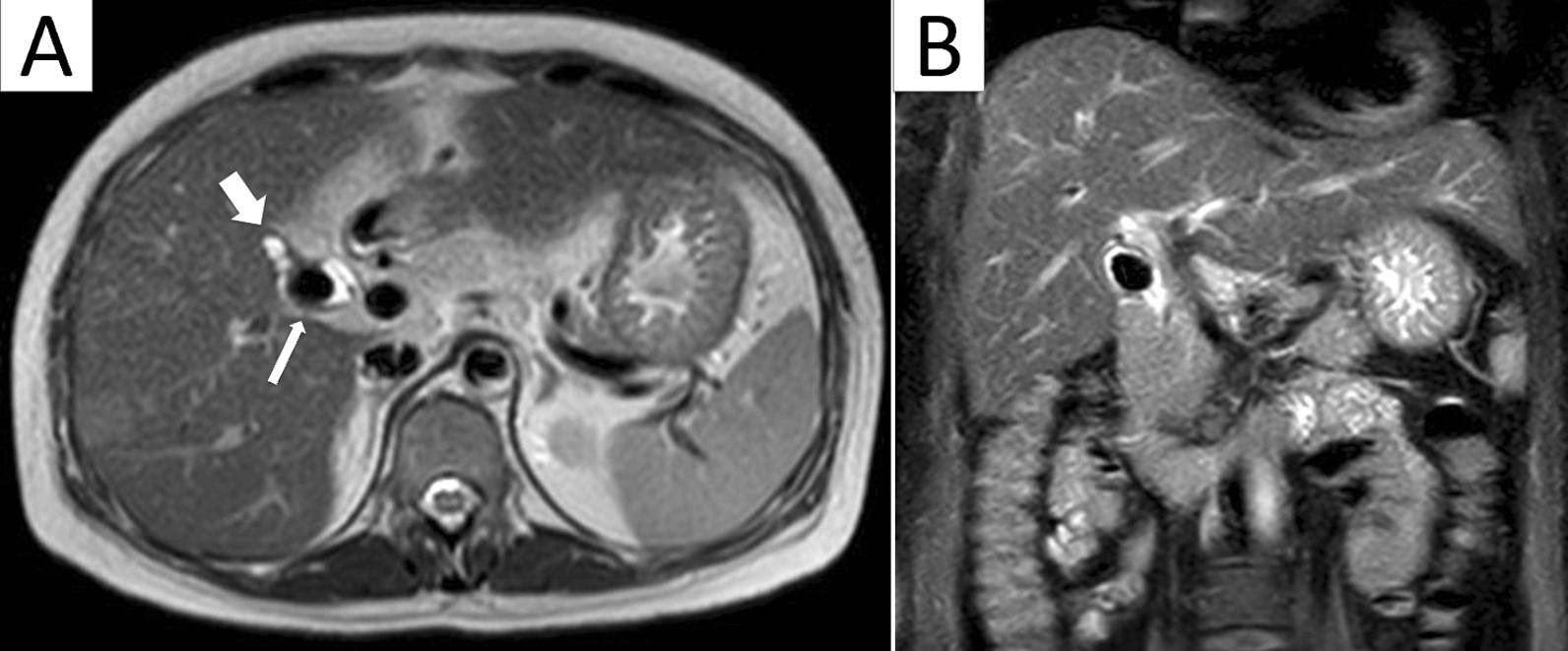
**A** a 2 × 2 cm stone (thin white arrow) impacted between the cystic duct and common bile duct (CBD). The thick white arrow indicates the severely contracted gall bladder. **B** Coronal view revealed the impacted stone causing compression of the CBD

A 45-year-old woman with poorly controlled diabetes mellitus presented to the emergency department with right upper abdomen tenderness and jaundice for 2 days. Lab examination revealed leukocytosis (white cell count: 13.5 × 10^3^) and elevated serum bilirubin and liver enzyme (total bilirubin 8.0 mg/dl, alkaline phosphatase 333 U/L). An abdominal echography revealed a gallbladder stone with a dilated common bile duct. Magnetic resonance cholangiopancreatography (MRCP) showed a contracted gallbladder with a 2 × 2 cm-sized stone impacted in the junction between the cystic duct and common bile duct (CBD) area causing compression of the CBD (Fig. 1). Type II Mirizzi syndrome (McSherry classification) was diagnosed. Endoscopic retrograde cholangiopancreatography (ERCP) was arranged for relieving jaundice. Meanwhile, one plastic stent was placed. Four weeks later, after the patient’s jaundice and gallbladder inflammation fully recovered, we proceeded with robotic-assisted cholecystectomy.

**Fig. 2:**
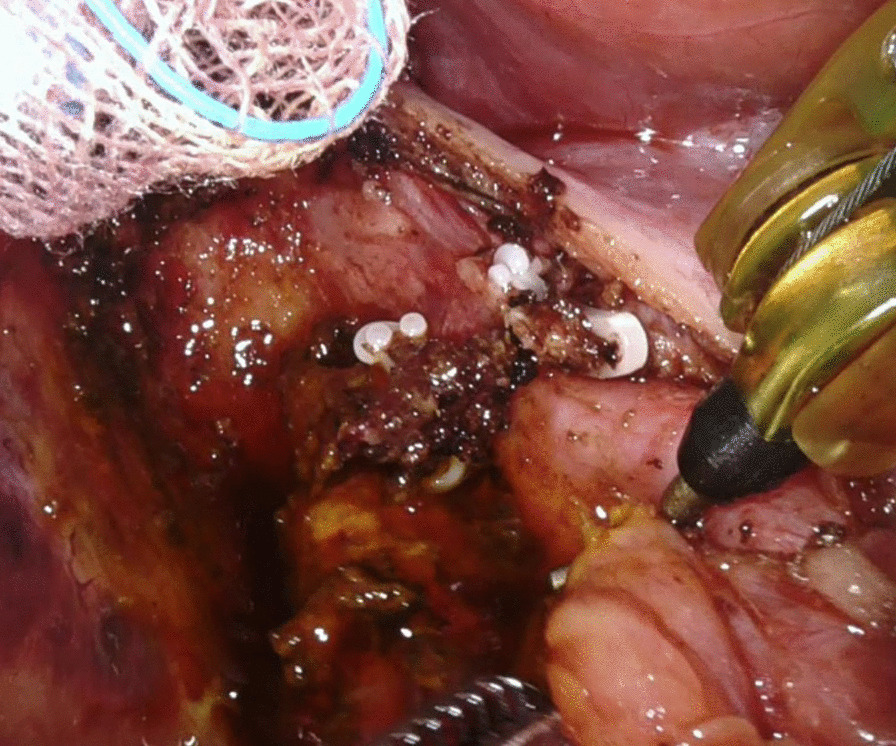
The proximal and distal stump of the posterior branch of the right hepatic artery were controlled by Hem-o-lok

During the operation, type II Mirizzi syndrome was confirmed. A partial cholecystectomy was performed with stone retrieval and closure of the cholecystobiliary fistula. Owing to unclear anatomy and severe adhesion over the Calot triangle, the anterior branch of the right hepatic artery was damaged. The proximal and distal stump of the right hepatic artery branch was then controlled by Hem-o-lok with mild intraoperative bleeding (Fig. 2). The postoperative course was uneventful, and the patient was discharged on day 6 after the operation.

**Fig. 3:**
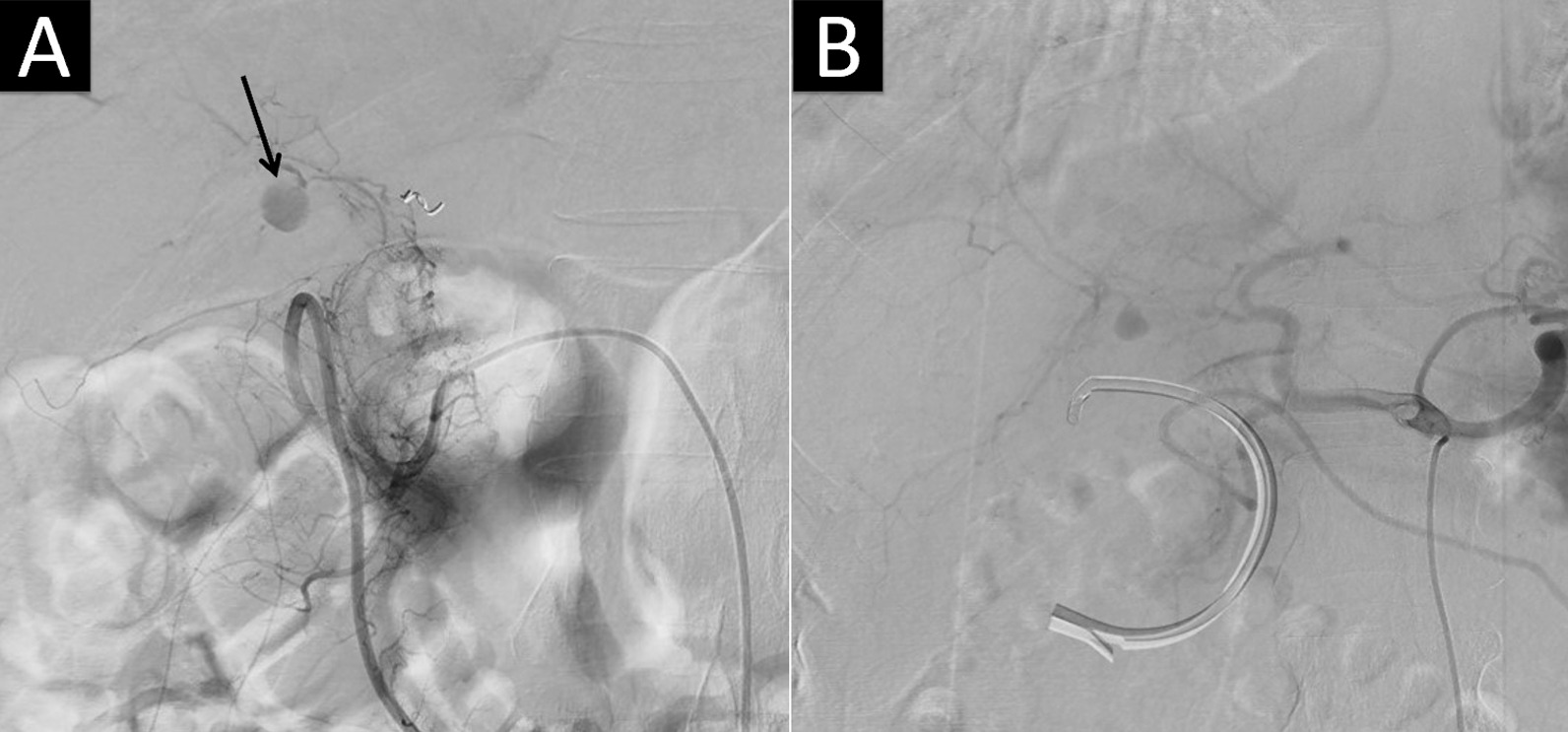
**A** The first angiogram revealed a 3 cm pseudoaneurysm (black arrow) located in the distal stump of the right hepatic artery anterior branch, supplied by the posterior branch of the right hepatic artery due to rich anastomosis. **B** The second angiogram revealed a recurrent pseudoaneurysm located in the same region, supplied by the GDA collateral vessels

On the 7th day after discharge, she presented in the emergency department again with right upper abdomen tenderness, melena, and jaundice. A panendoscopy revealed fresh blood coming from the ampulla of Vater. The total bilirubin level elevated to 9 mg/dl. ERCP revealed a blood clot inside the common bile duct without any biliary leakage. A computed tomography angiography (CTA) showed a 3 cm pseudoaneurysm located in the distal stump of the right hepatic artery anterior branch, supplied by the anastomosis from the posterior branch of the right hepatic artery (Fig. 3A). Then TAE was done via the posterior branch of the right hepatic artery using gelfoam material. No more pseudoaneurysm was found by angiography after embolization. Unfortunately, 3 days after TAE, the patient suffered from a sudden onset of abdominal pain. A panendoscopy revealed active bleeding from the ampulla of Vater. A new angiography showed a recurrent pseudoaneurysm at the same location with afferent artery from the branch of the gastroduodenal artery (GDA) (Fig. 3B). After several attempts, microcoil embolization was successfully performed via the GDA branch. The patient did not complain of any discomfort during the rest of the hospitalization and liver enzymes returned to normal. She was discharged one week after the second TAE. After 6 months of follow-up, the patient did not feel any discomfort and CTA revealed no further right hepatic artery pseudoaneurysm.

## Discussion and conclusions

Iatrogenic injury to the hepatic artery may be due to the intervention procedures or surgery of the hepatic hilar region [1]. Although iatrogenic hepatic artery pseudoaneurysm following laparoscopic cholecystectomy operation is a rare complication, a search of the literature reveals many case reports or case series [2–16]. The mechanism of hepatic artery pseudoaneurysm following laparoscopic cholecystectomy is unclear, but it is usually regarded as a result of direct damage of the vascular wall by inadequate grasping force or thermal injury [1], erosion due to improper clip position [16, 17], bile duct injury with bile leak, or biloma and infection [6]. In our case, the proximal and distal stump of the right hepatic artery branch was controlled by Hem-o-lok. But vessel injury may still occur in the intima or outer adventitia due to excessive vessel manipulation or inadequate hemostasis by a monopolar or bipolar electrocautery device. Damage of the arterial wall along with a strong backflow of collateral circulation may result in the formation of a pseudoaneurysm. A similar iatrogenic pseudoaneurysm formation following laparoscopic cholecystectomy can also occur in the cystic artery or gastroduodenal artery branch [18–20].

The clinical presentation of hepatic artery pseudoaneurysm includes intraperitoneal rupture, hemobilia due to rupture into the biliary tract, or jaundice due to compression of the bile duct [21]. Among these, the most common presenting symptom is hemobilia. The typical symptom of hemobilia is Quincke’s triad which comprises gastrointestinal bleeding, right upper abdomen tenderness, and jaundice. But only 20–40% of patients present with all the symptoms [5]. In our case, clinical jaundice was noted with minimal melena. The diagnosis of hemobilia was confirmed by endoscopy which revealed the blood directly from the orifice of the ampulla of Vater. Owing to the rare incidence but high severity due to rupture of the pseudoaneurysm, if abdomen tenderness or jaundice after laparoscopic cholecystectomy operation is noted, hemobilia should be considered. CTA and following TAE should be aggressively arranged for diagnosis and treatment [22]. Prompt and early recognition is necessary because the patient may be exsanguinated from the rupture of the pseudoaneurysm [23].

The etiology of hemobilia may be related to the formation of an arterio-biliary fistula and subsequent rupture of the pseudoaneurysm into the bile duct [24]. Other causes of hemobilia include trauma, liver biopsy, endoscopic retrograde cholangiopancreatography (ERCP), biliary stent placement, vasculitis, or hepatobiliary tumor [25–28]. The mean period between the initial laparoscopic cholecystectomy and diagnosis of hepatic artery pseudoaneurysm is 2 months (range 0.2–19 months) [1], but the initial clinical symptoms may occur within the first week after laparoscopic cholecystectomy [7].

The most appropriate treatment of hepatic artery pseudoaneurysm is TAE, which has a clinical success rate of around 85% [1, 3]. With few exceptions, recurrent pseudoaneurysm bleeding may occur despite the initial success of TAE. The rebleeding episode usually happens around 1 week after the first TAE [3]. Rarely, if embolization failed, surgery is the final way to ligate the pseudoaneurysm [2, 7]. The etiology of rebleeding of the hepatic artery pseudoaneurysm after TAE was unclear. Incomplete arterial occlusion, rich anastomosis between the left and right hepatic artery, and sizable multiple feeders of the pseudoaneurysm may be possible reasons for rebleeding after initial successful TAE [3, 28, 29]. The choice of embolization materials is mostly dependent on the operator’s clinical experience and material availability. In our case, the first embolization material was gelfoam instead of coils. Although the embolization was successful initially, pseudoaneurysm bleeding soon recurred due to multiple collateral anastomosis from the gastroduodenal artery which was not revealed in the first TAE. After the second TAE using microcoil material via the gastroduodenal artery, the pseudoaneurysm was permanently embolized. Generally, the use of steel coils as an embolization material for hepatic artery pseudoaneurysm is recommended. Although there seems to be no significant clinical success rate difference between the embolic materials [3].

The patient in this case report was diagnosed with type II Mirizzi syndrome before we proceeded to the laparoscopic operation. Although some literature suggests that a laparotomy operation is a preferred choice in type II Mirizzi syndrome, minimally invasive modality, including laparoscopy and robotic-assisted system, may be feasible and safe in the treatment of type II Mirizzi syndrome [30–34]. A robotic surgical system can provide a stable three-dimensional view and excellent manipulation via the endo-wrist instrument, which made controlling vessels and preventing the damage of the bile duct easier than traditional laparoscopy. However, despite the improvement of surgical instruments, when dealing with distorted anatomy or an adhesion condition of either Mirizzi syndrome or chronic cholecystitis, a careful preoperative diagnosis and lower conversion threshold to laparotomy before damaging the great vessel or bile duct may be an adequate policy.

In conclusion, hepatic artery pseudoaneurysm is a rare complication following laparoscopic cholecystectomy. Using advanced technology such as robotic surgical systems, adequate manipulation, and careful ligation of the vessel stump are important factors for preventing possible complications. Meanwhile, treatment of the pseudoaneurysm by embolization using a coil material may be a suitable method for preventing its recurrence.

## Data Availability

Data sharing does not apply to this article, as no datasets were generated or analyzed during the current study.

## Abbreviations

TAE: Trans-arterial embolization
CTA: Computed tomography angiography
MRCP: Magnetic resonance cholangiopancreaticography
CBD: Common bile duct
ERCP: Endoscopic retrograde cholangiopancreaticography
GDA: Gastroduodenal artery
